# Effects of Short-Term Corticosteroid Use on Reactogenicity and Immunogenicity of the First Dose of ChAdOx1 nCoV-19 Vaccine

**DOI:** 10.3389/fimmu.2021.744206

**Published:** 2021-09-22

**Authors:** Jinyoung Yang, Jae-Hoon Ko, Jin Yang Baek, Jinyeong Hong, Soyoung Ha, Beomki Lee, Kyungmin Huh, Sun Young Cho, Cheol-In Kang, Doo Ryeon Chung, Yae-Jean Kim, Eun-Suk Kang, Kyong Ran Peck

**Affiliations:** ^1^Division of Infectious Diseases, Department of Medicine, Samsung Medical Center, Sungkyunkwan University School of Medicine, Seoul, South Korea; ^2^Asia Pacific Foundation for Infectious Diseases (APFID), Seoul, South Korea; ^3^Department of Laboratory Medicine and Genetics, Samsung Medical Center, Sungkyunkwan University School of Medicine, Seoul, South Korea; ^4^Division of Infectious Diseases and Immunodeficiency, Department of Pediatrics, Samsung Medical Center, Sungkyunkwan University, School of Medicine, Seoul, South Korea

**Keywords:** reactogenicity, immunogenicity, COVID-19, vaccine, steroid

## Abstract

The effects of corticosteroid use on the reactogenicity and immunogenicity of ChAdOx1 nCoV-19 (ChAd) vaccine were evaluated. Healthcare workers (HCWs) who took low-dose corticosteroid agents around the time of the first dose of ChAd (ChAdPd group) were recruited and the reactogenicity and immunogenicity were compared with those of ChAd (ChAd group) and BNT162b2 vaccination (BNT group) of HCWs without corticosteroid exposure. The immunogenicity was measured three weeks after vaccination using quantitative anti-SARS-CoV-2 spike protein (S) antibody electrochemiluminescence immunoassay and interferon gamma (IFN-γ) release assay. A total of 67 HCWs comprising 24 ChAd, 29 BNT, and 14 ChAdPd was included. The median total corticosteroid dose of the ChAdPd group was 30 mg prednisolone equivalents (interquartile range (IQR) 20–71.3 mg). HCWs in the ChAdPd group experienced significantly milder reactogenicity (median total score 7.5, IQR 4.0–18.0) compared to those in the ChAd group (median 23.0, IQR 8.0–43.0, *P*=0.012) but similar to that in the BNT group (median 5.0, IQR 3.0–9.0, *P*=0.067). The S antibody concentration of the ChAdPd group (62.4 ± 70.0 U/mL) was higher than that of the ChAd group, though without statistical significance (3.45 ± 57.6 U/mL, *P*=0.192). The cellular immune response was most robust in the ChAdPd group, with significantly higher IFN-γ concentration (5.363 ± 4.276 IU/mL), compared to the ChAd (0.978 ± 1.181 IU/mL, *P*=0.002) and BNT (1.656 ± 1.925 IU/mL, *P*=0.009) groups. This finding suggest that short-term corticosteroid reduces reactogenicity of the first dose of ChAd without hindering immunogenicity.

## Introduction

To address the coronavirus disease 2019 (COVID-19) pandemic, vaccines against severe acute respiratory syndrome coronavirus 2 (SARS-CoV-2) have been developed in an unprecedentedly brief period (1, 2). BNT162b2 (BNT), an mRNA-based vaccine (BioNTech/Pfizer), was approved for conditional marketing authorization by the European Medicines Agency (EMA) on December 2020, followed by ChAdOx1 nCoV-19 (ChAd), a chimpanzee adenovirus-vectored vaccine (Oxford/AstraZeneca) on January 2021 (3, 4). However, unpredictably higher reactogenicity after the first dose of ChAd compared to that of BNT was observed in the real-world vaccinations (5, 6). To control post-vaccination fever or local reactions, healthcare authorities recommend acetaminophen (AAP), but it remains unknown how anti-inflammatory agents affect the immunogenicity of COVID-19 vaccines (5–7). There is little evidence regarding the effect of corticosteroid, a potent anti-inflammatory agent, on the immunogenicity of the COVID-19 vaccine, which would be essential data to guide patients on corticosteroid use (8).

During mass vaccination of healthcare workers (HCWs) with ChAd, several HCWs who used corticosteroid agents for various reasons experienced lower reactogenicity than those who did not. To investigate the immunogenicity of HCWs with corticosteroid exposure around the time of the first dose of ChAd, we measured humoral and cellular immunity using quantitative anti-SARS-CoV-2 S antibody assay and SARS-CoV-2 specific interferon gamma (IFN-γ) release assay (IGRA) at three weeks after vaccination.

## Methods

### Study Design

A prospective cohort study evaluating post-vaccination reactogenicity and immunogenicity was conducted in the Republic of Korea, at a 1950-bed tertiary care hospital which has more than 5,000 HCWs. According to the COVID-19 vaccination policy of the Korean government, most HCWs were vaccinated with ChAd, although 200 HCWs who were designated for COVID-19 patient care were vaccinated with BNT. The first doses of ChAd were administered between March and May 2021, and the second dose occurred 10 to 12 weeks after the first dose. The first and second BNT vaccinations were administered during March 2021, with a three-week interval between them. HCWs who took low-dose corticosteroid agents (oral prednisolone or methylprednisolone) around the time of the first dose of ChAd were recruited (ChAdPd group), and their reactogenicity and immunogenicity were compared with those of ChAd- and BNT-vaccinated HCWs without corticosteroid exposure (ChAd group and BNT group, respectively). For comparison of the cellular immune response of vaccinated HCWs and SARS-CoV-2-infected individuals, IGRA results of 10 convalescent COVID-19 patients were used.

### Heterogeneous Boosting of ChAdPd Group With BNT

According to the vaccination policy of Korean government, HCWs in ChAdPd group received heterogeneous boosting with BNT, 12 weeks after the first ChAd vaccination. HCWs in ChAd group finished homogenous boosting with ChAd before the administration of heterogeneous boosting policy. Since no one took corticosteroid in ChAd group around the second dose of vaccination while half of ChAdPd group took short-term corticosteroid around the heterogeneous boosting, we conducted following reactogenicity and immunogenicity investigation among ChAdPd group.

### eDiary for Reactogenicity

The reactogenicity data after the first dose of vaccination were collected for seven days using an electronic diary (eDiary) format, which was developed based on phase III clinical trials of the vaccines (1, 2). A total of 11 side effects as well as the need for AAP to control side effects were investigated. Local side effects included pain, redness, and swelling at the injection site. Systemic side effects were fever, chill, myalgia, arthralgia, fatigue, headache, vomiting, and diarrhea. Participants rated each symptom on a scale of 0 to 4 every day from Day 0 (vaccination day) to Day 7. If there were no symptoms, a score of 0 was selected, 1 for mild, 2 for moderate, 3 for severe, and 4 for critical. For AAP, a score of 0 was selected for no need for AAP, 1 for 1–2 tablets per day, 2 for 3–4 tablets, 3 for 5–6 tablets, and 4 for more than 7 tablets. Information about age, sex, underlying diseases, body mass index (BMI), and any medications taken within 1 week of vaccination also were collected.

### Laboratory Procedures

#### Anti-SARS-CoV-2 Nucleocapsid Antibody Test

To investigate undiagnosed previous SARS-CoV-2 infection, qualitative anti-SARS-CoV-2 nucleocapsid antibody was measured using Elecsys^®^ Anti-SARS-CoV-2 (Roche Diagnostics, Basel, Switzerland). Because nucleocapsid is not contained in the SARS-CoV-2 vaccines, the presence of anti-nucleocapsid antibody suggests previous SARS-CoV-2 infection, rather than vaccine-induced immunity (9). A recombinant nucleocapsid protein was used for the detection of high-affinity antibodies against SARS-CoV-2 (10). A double-antigen sandwich principle was utilized and the electrochemiluminescence immunoassay (ECLIA) method was applied using cobas e immunoassay analyzers. The detectable isotypes included IgA and IgG (11). A cut-off index (COI) greater than or equal to 1.0 was considered positive.

#### Quantitative Anti-SARS-CoV-2 Spike Protein Antibody Assay

For the quantitative measurement of post-vaccination humoral immune response, a quantitative anti-SARS-CoV-2 spike protein antibody test kit (Elecsys^®^ Anti-SARS-CoV-2 S, Roche Diagnostics) was used (12). The kit was developed for *in vitro* quantitative measurement of anti-SARS-CoV-2 spike protein antibodies with the ECLIA method using cobas e analyzers. A recombinant receptor binding domain of spike protein was used with a double-antigen sandwich principle. While the antigen used in the kit was captured predominantly by IgG, IgA and IgM also were detectable. An anti-SARS-CoV-2 S antibody concentration ≥0.8 U/mL was considered positive. The linear range was 0.4–250 U/mL, and automated dilution was performed in the cobas e analyzers.

#### SARS-CoV-2 Specific Cellular Immunity Test

We investigated cell-mediated immunity by measuring IFN-γ secreted by T cells in response to the SARS-CoV-2 antigen, using a SARS-CoV-2 specific IGRA kit with enzyme-linked immunosorbent assay (ELISA) (Covi-FERON ELISA, SD Biosensor, Suwon, Republic of Korea). Whole blood specimens from the participants were collected, and 1 mL was injected into each Covi-FERON tube (Nil tube, SARS-CoV-2 spike protein antigen (Sp)1 tube, Sp2 tube, and Mitogen tube). The Sp1 tube contained spike protein antigens derived from the original SASR-CoV-2 (Wuhan/Hu-1/2019) and B.1.1.7 variant, while the Sp2 tube contained those derived from the B.1.351 and P.1 variants (13). After incubating at 37°C for 16–24 h, plasma was collected by centrifuging the tubes for 15 min at 2200–2300g. IFN-γ was detected by ELISA and the measured optical density was converted to IFN-γ concentration (IU/mL) using ELISA Report Software (SD Biosensor). Because the positive cut-off value of the SARS-CoV-2 IGRA kit had not been established, IFN-γ concentration of the Sp tubes minus that of the Nil tube was compared quantitatively between the groups, and a tentative cut-off values were applied: mean + 3 standard deviations (SDs) of IFN-γ concentration of the Nil tubes (0.84 IU/mL), which is higher than that used in previous IGRAs for tuberculosis (0.35 IU/mL) or CMV (0.2 IU/mL) (14, 15).

### Statistical Analysis

To compare the characteristics, reactogenicity, and laboratory test results of the vaccinated groups, either Student’s *t*-test or Mann–Whitney U test was used for continuous variables and the Chi-square or Fisher’s exact test for categorical variables. All *P*-values were two-tailed and those <0.05 were considered statistically significant. IBM SPSS Statistics version 27 (IBM, Armonk, NY, USA) was used for all statistical analyses.

## Results

### Baseline Characteristics and Reactogenicity of Vaccinated HCWs

A total of 67 HCWs comprising 24 ChAd, 29 BNT, and 14 ChAdPd was included in the present analysis. The median age of the enrolled HCW was 35 years (interquartile range (IQR) 28.0–46.0), and those in the BNT group were younger than those in the other two groups (**Table 1**). Most HCWs were female (n = 51, 76.1%) and had low BMI (median 21.0, IQR 20.1–23.3). HCWs in the ChAdPd group took oral prednisolone (5 mg tablet) or methyl prednisolone (4 mg tablet) as 1 or 2 tablets twice a day or 2 tablets three times a day for up to five days. The total cumulative dose was 30 mg prednisolone equivalents in median (IQR 20–71.3 mg). They took corticosteroid as prescribed by a doctor to control underlying disease activity (allergic rhinitis) or to avoid adverse effects of ChAd.

**Table 1:** Baseline characteristics, reactogenicity, and immunogenicity of vaccinated HCWs.

Variables	ChAd (n = 24)	BNT (n = 29)	ChAdPd (n = 14)	*P* value		
				ChAd vs. BNT	ChAd *vs.* ChAdPd	BNT *vs.* ChAdPd
**Demographics**						
Age, year	36.5 (34.3–48.8)	28.0 (26.0–33.0)	44.0 (39.5–49.3)	**<0.001**	0.161	**<0.001**
Male	6 (25.0)	8 (27.6)	2 (14.3)	1.000	0.435	0.333
BMI, kg/m^2^	21.1 (19.9–22.7)	21.0 (19.9–23.9)	21.7 (20.1–23.7)	0.796	0.463	0.586
Underlying diseases, any^*^	2 (8.3)	4 (13.8)	2 (14.3)	0.532	0.564	0.965
**Anti-inflammatory drugs^†^**						
NSAID	2 (8.3)	0 (0.0)	0 (0.0)	NA	NA	NA
Corticosteroid	0 (0.0)	0 (0.0)	14 (100.0)	NA	NA	NA
**Reactogenicity, score sum**						
**Acetaminophen use**	1.0 (0.0–3.0)	0.0 (0.0–0.0)	0.0 (0.0–0.0)	**0.001**	**0.005**	0.150
***Local***						
Pain	4.5 (2.3–7.0)	3.0 (1.0–4.0)	4.0 (3.0–4.8)	**0.003**	0.687	**0.015**
Redness	0.0 (0.0–0.0)	0.0 (0.0–0.0)	0.0 (0.0–0.0)	0.116	0.754	0.675
Swelling	0.0 (0.0–0.0)	0.0 (0.0–0.0)	0.0 (0.0–0.5)	0.967	0.731	0.580
***Systemic***						
Fever	0.0 (0.0–0.0)	0.0 (0.0–0.0)	0.0 (0.0–0.0)	**0.011**	0.463	0.150
Chill	2.5 (1.0–5.0)	0.0 (0.0–0.0)	0.0 (0.0–0.0)	**<0.001**	**0.001**	0.570
Myalgia	4.5 (0.0–6.0)	0.0 (0.0–2.0)	0.0 (0.0–2.25)	**0.002**	0.054	0.953
Arthralgia	0.0 (0.0–4.8)	0.0 (0.0–0.0)	0.0 (0.0–0.0)	**0.001**	0.120	0.218
Fatigue	3.5 (1.3–7.8)	0.0 (0.0–1.5)	2.5 (0.0–4.3)	**<0.001**	0.212	0.066
Headache	3.0 (1.3–7.8)	0.0 (0.0–0.5)	0.0 (0.0–2.0)	**<0.001**	**0.001**	0.332
Vomiting	0.0 (0.0–0.0)	0.0 (0.0–0.0)	0.0 (0.0–0.0)	0.272	0.846	1.000
Diarrhea	0.0 (0.0–0.0)	0.0 (0.0–0.0)	0.0 (0.0–0.0)	0.503	0.410	0.218
***Total score***	23.0 (8.0–43.0)	5.0 (3.0–9.0)	7.5 (4.0–18.0)	**<0.001**	**0.012**	0.067
**Anti-SARS-CoV-2 N ab**						
COI value	0.09 ± 0.01	0.11 ± 0.14	0.10 ± 0.03	NA	NA	NA
Positivity, %	0 (0.0)	0 (0.0)	0 (0.0)	NA	NA	NA
**Anti-SARS-CoV-2 S ab**						
Concentration, U/mL	3.45 ± 57.6	108.7 ± 64.8	62.4 ± 70.0	**<0.001**	0.192	**0.038**
Positivity, %	23 (95.8)	29 (100.0)	14 (100.0)	0.267	0.439	NA
SARS-CoV-2 IGRA^*^						
Nil, IU/mL	0.189 ± 0.125	0.240 ± 0.435	0.183 ± 0.271	0.726	0.946	0.695
Sp1 - Nil, IU/mL	0.978 ± 1.181	1.656 ± 1.925	5.363 ± 4.276	0.377	**0.002**	**0.009**
Sp2 - Nil, IU/mL	0.816 ± 1.498	0.878 ± 0.608	3.149 ± 3.249	0.904	**0.029**	**0.023**
Mitogen - Nil, IU/mL	13.859 ± 0.706	14.119 ± 0.577	14.193 ± 0.308	0.379	0.127	0.684
**Tentative positive cut-offs**						
*Sp1 - Nil > 0.84 IU/mL*	4 (40.0)	6 (60.0)	13 (92.9)	0.656	**0.005**	0.051
*Sp2 - Nil > 0.84 IU/mL*	3 (30.0)	5 (50.0)	9 (64.3)	0.361	0.214	0.484

Data are expressed as the number (%) of HCWs or median (IQR). Statistically significant P values are presented as bold texts. ^*^Underlying diseases included migraine, pituitary adenoma, endometriosis, Grave’s disease, arrhythmia, hypertension, hypothyroidism, and allergic rhinitis. ^†^Taken within one week from vaccination (before and after). One HCW took three tablets of ibuprofen and the other had one tablet of naproxen. HCWs in the ChAdPd group took oral prednisolone (5 mg tablet) or methyl prednisolone (4 mg tablet) 1t bid, 1t tid, or 2t bid for up to five days. The total cumulative dose was 30 mg prednisolone equivalents in median (IQR 20–71.3 mg). ^*^IGRA was performed in 10 ChAd, 10 BNT, and 14 ChAdPd HCWs. Because the positive cut-off value of SARS-CoV-2 IGRA had not been established, a tentative cut-off values were applied: mean + 3 standard deviation of IFN-γ concentration of the Nil tubes (0.84 IU/mL).

HCW, healthcare worker; ChAd, ChAdOx1 nCoV-19-vaccinated group; BNT, BNT162b2-vaccinated group; ChAdPd, corticosteroid-exposed ChAdOx1 nCoV-19-vaccinated group; BMI, body mass index; NSAID, non-steroidal anti-inflammatory drug; NA, not applicable; COI, cut-off index; N, nucleocapsid; ab, antibody; S, spike protein; IGRA, interferon-gamma releasing assay; Sp, spike protein antigen.

**Figure 1:**
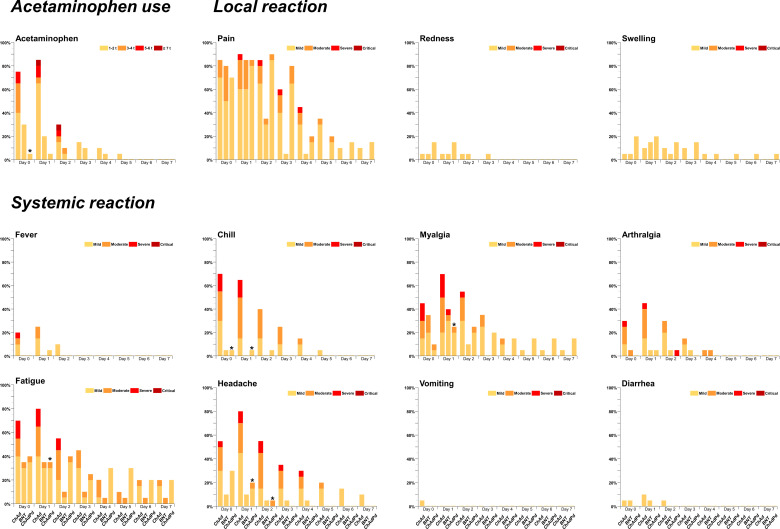
Daily reactogenicity score of the ChAd, BNT, and ChAdPd groups. A total of 11 side effects and the need for AAP to control side effects were investigated and presented as a numeric score of 0 to 4. The asterisk (*) denotes significantly reduced side effects in the ChAdPd group compared to the ChAd group. HCWs took AAP to control side effects from 1 tablet once a day to 2 tablets three times a day. One HCW ChAd group took 2 tablets four times a day on day 1 and 2 to control severe headache. t, tablet; AAP, acetaminophen; ChAd, ChAdOx1 nCoV-19-vaccinated group; BNT, BNT162b2-vaccinated group; ChAdPd, corticosteroid-exposed ChAdOx1 nCoV-19-vaccinated group.

HCWs in the ChAd group experienced significantly higher reactogenicity (total score in median 23.0, IQR 8.0–43.0) compared to those in the BNT group (total score in median 5.0, IQR 3.0–9.0; *P* < 0.001). The reactogenicity scores for pain, fever, chill, myalgia, arthralgia, fatigue, headache, and AAP requirement were significantly higher in the ChAd group compared to the BNT group (all *P* < 0.05). The reactogenicity scores were relatively lower in the ChAdPd group than in the ChAd group. In the comparison between the ChAdPd and BNT groups, only the pain score was significantly higher in the ChAdPd group (*P* = 0.015). When the scores were compared between the ChAd and ChAdPd groups, the total reactogenicity scores were significantly lower in the ChAdPd group (median 7.5, IQR 4.0–18.0; *P* = 0.012). HCWs in the ChAdPd group experienced less chill, fatigue, myalgia, headache, or AAP requirement for both the overall and daily scores (**Figure 1**).

### Immunogenicity of Vaccinated HCWs at the Third Week of Vaccination

Anti-SARS CoV-2 nucleocapsid antibody was measured to investigate undiagnosed previous SARS-CoV-2 infections, and all participating HCWs showed negative results (**Table 1**). In the assessment of humoral antibody response in the vaccinated HCWs using quantitative anti-SARS-CoV-2 S antibody test kit, all showed positive test result except one in the ChAd group. The S antibody concentrations were significantly higher in the BNT group (108.7 ± 64.8), compared to the ChAd group (3.45 ± 57.6, *P* < 0.001) and the ChAdPd group (62.4 ± 70.0, *P* = 0.038). The S antibody concentrations of ChAdPd group were higher than those of the ChAd group, without statistical significance (*P* = 0.192).

Cellular immune responses of the vaccinated HCWs was measured in 10 ChAd, 10 BNT, and 14 ChAdPd HCWs using SARS-CoV-2 specific IGRA test. Baseline IGRA was tested in the ChAd and BNT groups, and all of the tests were negative (-0.020 ± 0.051 for Sp1 minus nil and -0.030 ± 0.076 for Sp2 minus nil). In the overall cohort at the third week of vaccination, 23 HCWs (67.7%) showed a positive test result with Sp1 based on a tentative cut-off value of 0.84 IU/mL. The cellular immune responses to Sp1 and Sp2 were most robust in the ChAdPd group, showing a significantly higher IFN-γ concentration compared to the ChAd and BNT groups (all *P* < 0.05). The proportion of positive results was also highest in the ChAdPd group, while the cellular immune response between the ChAd and BNT groups was similar both in IFN-γ concentrations and positive proportion of tests.

The cellular immune response of the ChAdPd group was compared to that of 10 COVID-19 patients, comprising three mild cases (required O2 supplement *via* nasal prong) and seven severe cases (required O2 supplement *via* high flow nasal cannula). An IGRA was performed on COVID-19 patients in the convalescence phase (median 74, IQR 59.3–128.5 days from symptom onset). The IFN-γ concentrations of the HCWs in the ChAdPd group were higher than those of the convalescent COVID-19 patients, without statistical significance (**Supplementary Table 1**).

### Reactogenicity and Immunogenicity of ChAdPd Group After Heterogeneous Boosting With BNT

Among HCWs in ChAdPd group, 12 received heterogeneous boosting with BNT, after 12 weeks of the first vaccination according to the vaccination policy of Korean government. Seven HCWs took oral corticosteroids as 1 or 2 tablets twice a day or 1 tablets three times a day for up to three days. The total cumulative dose was 20 mg prednisolone equivalents in median (IQR 10–30 mg). HCWs who took corticosteroid experienced lower reactogenicity (total score in median 3.0, IQR 2.0–7.0) compared to those who did not without statistical significance (total score in median 8.0, IQR 1.5–25.0; *P* = 0.326). Anti-SARS-CoV-2 S antibody titers of HCWs who took corticosteroid were slightly higher (7794.14 ± 3763.56) than those of HCWs who did not (6308.40 ± 4405.08), without statistical significance (*P* = 0.231). IFN-γ concentrations of HCWs who took corticosteroid were slightly lower (Sp1, 3.86 ± 2.41 IU/mL and Sp2, 1.82 ± 1.29 IU/mL) than those of HCWs who did not (Sp1, 5.67 ± 2.45 IU/mL; Sp2, 3.16 ± 1.94 IU/mL), without statistical significance (Sp1, *P* = 0.231 and Sp2, *P* = 0.179).

## Discussion

On March 2021, massive COVID-19 vaccination started among HCWs in the Republic of Korea, including BNT for HCWs designated for COVID-19 patient care and ChAd for most other HCWs. Because vaccine-induced immune thrombotic thrombocytopenia, a rare potentially fatal side effect of adenoviral vector vaccines, had not been reported by that time (16, 17), ChAd vaccination candidates included young HCWs who reported significantly more severe reactogenicity compared to ChAd-vaccinated older HCWs or BNT-vaccinated HCWs in similar age groups (5, 6). Although the Korea Disease Control and Prevention Agency restricted ChAd vaccination candidates to those over 30 years old from 12 April, 2021 (18), ChAd became known for potentially severe side effects and reactogenicity among HCWs. In addition to those with underlying allergic rhinitis, several HCWs took corticosteroid agents to avoid potential side effects, based on the experiences of alleviation of acute symptoms of upper respiratory tract infections (19). We enrolled 14 HCWs who took corticosteroid agents in the peri-vaccination period of the first dose of ChAd and evaluated humoral and cellular immune responses at the third week after vaccination.

Of note, HCWs in the ChAdPd group experienced remarkably lower reactogenicity compared to the HCWs in the ChAd group. Total reactogenicity scores of the ChAdPd group were significantly lower than those of the ChAd group and no one experienced severe or critical side effects. Humoral immune response was not compromised in the ChAdPd group, and average antibody concentration was higher in the ChAdPd group compared to the ChAd group. Moreover, cellular immune response measured by SARS-CoV-2 IGRA test was significantly stronger in the ChAdPd group than in the ChAd and BNT groups. IFN-γ concentration of the ChAdPd group was higher than that in the convalescing COVID-19 patients. Because of the small size of the study population, it is difficult to conclude an enhanced immune response of the ChAdPd group. However, our findings do indicate that short-term corticosteroid use during the peri-vaccination period of the first dose of ChAd did not hinder immunogenicity of the vaccine.

Generally, it is recommended to avoid corticosteroid agents in peri-vaccination periods because they can interrupt the immunogenicity of the vaccine. Observational studies conducted on the recipients of either the pneumococcal polysaccharide vaccine or the hepatitis B vaccine indicated that long-term steroid use can decrease serologic response (20, 21). On the other hand, it was suggested that short-term, high-dose steroid use did not affect the immunogenicity of the influenza vaccine (22, 23). The potential effect of corticosteroid use on the immunogenicity of COVID-19 vaccines has not been thoroughly investigated. There was a report that the antibody level was lower in a low-dose steroid user in an older adult cohort who received two doses of mRNA vaccine, but the sample size was small and statistical significance was not achieved (24). It also was reported that the immunogenicity of COVID-19 vaccines in solid organ transplant recipients was poor, but they took T-cell suppressive agents in addition to corticosteroids (9, 25). In the present study, we observed that short-term use of a low-dose corticosteroid in the peri-vaccination periods of ChAd did not hinder antibody response, while IFN-γ response in the ChAdPd group was comparable to or stronger than that of its comparators. One potential hypothesis of this phenomenon is that by inhibiting acute immune response against vector adenovirus, the delivery of DNA in the vector adenovirus to host cells could be more effective. Although the reason why the first dose of ChAd provokes more severe reactogenicity compared to the first dose of BNT has not been identified, acute immune response against the vector adenovirus is a plausible reason (6). It has been reported that systemic administration of adenovirus as a gene transfer vector induces innate, pro-inflammatory immune response (26, 27). An animal study exhibited that dexamethasone pre-treatment reduced innate and adaptive immune response to the adenovirus vector without reducing efficacy of gene transduction (27). As a following investigation, we conducted single cell transcriptome sequencing in healthy adults vaccinated with ChAd and noticed immediate monocyte activation occurs from the next day of vaccination (unpublished data). The increased activity of monocytes waned in the following specimens taken five and 12 days after vaccination. Further investigation about immediate immune response among ChAdPd group and after the second dose of ChAd is currently ongoing. In addition, we evaluated effect of short-term corticosteroid use among ChAdPd group after the heterogeneous boosting with BNT. The humoral and cellular immunogenicity was not significantly different between HCWs who took short-term corticosteroid and those who did not. Although experimental data explaining possible mechanism of the present study findings have not been fully elucidated yet, we noticed that short-term corticosteroid use in peri-vaccination period does not hinder immunogenicity of COVID-19 vaccines. The findings and hypothetical mechanisms of the present study need to be investigated in detail by following studies.

Our study had several limitations. First, the number of participants was small and they were confined to relatively young and healthy HCWs. Meanwhile, since high reactogenicity of ChAd is most significant among young age groups, the present observation in young and healthy HCWs would be meaningful. In an observational study, 48.4% of HCWs aged 20–29 experienced fever after the first dose of ChAd, but only 12.2% of HCWs aged ≥ 50 did (5). Effect of short-term corticosteroid use on the immunogenicity of ChAd in old age group need to be evaluated, it might not have significant effect on the reactogenicity of that group. Second, the doses of corticosteroid were heterogeneous. Third, immunogenicity of the participants was mainly evaluated after the first vaccination dose. Although we investigated effect of heterogeneous booster by BNT of the ChAdPd group, the evaluated number of HCWs was limited. Fourth, IGRA has not been validated for COVID-19. However, IGRA is a well-validated method in the evaluation of latent tuberculosis, and its application for the evaluation of cell-mediated immunity of viral infections including cytomegalovirus is recently considered (28). For the evaluation of functionality of cell mediated immunity, detailed experiments such as SARS-CoV-2-specific T cell analysis using MHC class I multimer staining need be conducted (29). Fifth, anti-SARS-CoV-2 spike protein antibody test kit was used for the measurement of humoral response, while neutralization test was not conducted. In an ongoing investigation, we investigated titer correlation of the kit we used with plaque reduction neutralization test, and noticed statistically significant linearity between the titers of two methods (*R* = 0.76, *P* < 0.05; unpublished data). Lastly, the present study observation occurred in a special situation of an adenoviral vector vaccine, it cannot be generalized to other COVID-19 vaccines. Despite these limitations, our study findings suggest novel insights into the reactogenicity and immunogenicity of the adenovirus vector vaccine, eliciting further investigations.

## Conclusion

In conclusion, in an observational cohort study evaluating the immunogenicity of COVID-19 vaccines, short-term low-dose corticosteroid use in the peri-vaccination period of ChAd reduced reactogenicity without hindering immunogenicity.

## Data Availability Statement

The original contributions presented in the study are included in the article/**Supplementary Material**. Further inquiries can be directed to the corresponding authors.

## Ethics Statement

The studies involving human participants were reviewed and approved by the Institutional Review Board (IRB) of Samsung Medical Center. The patients/participants provided their written informed consent to participate in this study.

## Author Contributions

JY, J-HK, and KRP contributed to the conceptualization. JY, J-HK, JH, SH, KH, SYC, C-IK, and DRC contributed to the investigation. JYB, BL, and E-SK contributed to the laboratory work and methodology. Y-JK, E-SK, and KRP contributed to the supervision. JY, J-HK, E-SK, and KP contributed to the writing, review, and editing. All authors contributed to the article and approved the submitted version.

## Funding

This work was supported by Samsung Medical Center Grant #SMO1210321.

## Conflict of Interest

The authors declare that the research was conducted in the absence of any commercial or financial relationships that could be construed as a potential conflict of interest.

## Publisher’s Note

All claims expressed in this article are solely those of the authors and do not necessarily represent those of their affiliated organizations, or those of the publisher, the editors and the reviewers. Any product that may be evaluated in this article, or claim that may be made by its manufacturer, is not guaranteed or endorsed by the publisher.
