# Supplementary material for: Effects of Short-Term Corticosteroid Use on Reactogenicity and Immunogenicity of the First Dose of ChAdOx1 nCoV-19 Vaccine
Source: Front Immunol. 2021 Sep 22;12:744206. doi: 10.3389/fimmu.2021.744206 (PMC8493039; doi:10.3389/fimmu.2021.744206)
Supplement: Supplementary file 1 [file Table_1.docx]

**Supplementary Table 1. Cellular immune response of the ChAdPd group, compared to convalescing COVID-19 patients**

| Variables | COVID-19 patients  (n = 10) | ChAdPd  (n = 14) | *P* value |
| --- | --- | --- | --- |
| SARS-CoV-2 IGRA^*^ |  |  |  |
| Nil, IU/mL | 0.063 ± 0.020 | 0.183 ± 0.271 | 0.122 |
| Sp1 - Nil, IU/mL | 2.660 ± 2.481 | 5.363 ± 4.276 | 0.087 |
| Sp2 - Nil, IU/mL | 1.687 ± 1.672 | 3.149 ± 3.249 | 0.207 |
| Mitogen - Nil, IU/mL | 13.860 ± 1.123 | 14.193 ± 0.308 | 0.382 |
| Tentative positive cut-offs |  |  |  |
| *Sp1 - Nil > 0.84 IU/mL* | 8 (80.0) | 13 (92.9) | 0.348 |
| *Sp2 - Nil > 0.84 IU/mL* | 6 (60.0) | 9 (64.3) | 0.831 |

Data are expressed as the number (%) of persons or mean ± SD. COVID-19 patients included three mild cases (required O_2_ supplement via nasal prong) and seven severe cases (required O_2_ supplement via high flow nasal cannula). COVID-19 patients underwent IGRA at convalescence phase (median 74, IQR 59.3 – 128.5 days from symptom onset).

Abbreviations: COVID-19, coronavirus disease 2019; ChAdPd, corticosteroid-exposed ChAdOx1 nCoV-19-vaccinated group; IGRA, interferon-gamma releasing assay; Sp, spike protein antigen; SD, standard deviation
